# Foodborne botulism and the importance of recognizing the disease in the emergency department: a case report

**DOI:** 10.1186/s13256-023-03885-2

**Published:** 2023-04-15

**Authors:** Seppe De Vet, Thomas Tackaert, Mike Smet, Hannelore Raemen

**Affiliations:** 1 Department of Medicine and Pharmacy, Free University of Brussel, Brussels, Belgium; 2 Department of Medicine and Health Sciences, Ghent University, Ghent, Belgium; 3 Department of Emergency Medicine, Middelheim Hospital, Antwerp, Belgium

**Keywords:** Botulism, Myasthenic disorder, Trivalent botulinum antitoxin, HBAT, Case report

## Abstract

**Background:**

Botulism is a rare neuroparalytic disease that has only presented itself 19 times in the last 30 years in Belgium. Patients present to emergency services with a wide range of complaints. Foodborne botulism is a forgotten yet life-threatening disease.

**Case presentation:**

We describe a case of a Caucasian female in her 60s that presented to the emergency with reflux with nausea and spasmodic epigastric pain, no vomiting, dry mouth, and weakness in both legs. The symptoms started after ingestion of Atlantic wolffish. After exclusion of other more common causes, foodborne botulism was suspected. The patient was admitted to the intensive care unit for mechanical ventilation. Following treatment with trivalent botulinum antitoxin, she made a full neurologic recovery.

**Conclusion:**

It is important to rapidly recognize the possible diagnosis of botulism even if the neurological symptoms are not dominant. Rapid neurologic dysfunction and respiratory difficulties starts between 6 and 72 hours after ingestion. The decision to administer antitoxins should, however, be based on the presumptive clinical diagnosis and diagnosis should not delay therapy.

## Background

Botulism is a rare neuroparalytic disease caused by the neurotoxin botulinum, mainly produced by the *Clostridium botulinum* bacterium and less frequently by *Clostridium baratii* or *Clostridium butyricum*. *C. botulinum* is ubiquitous and easily isolated from the surfaces of vegetables, fruits, and seafood and exists in soil and marine sediment worldwide [1]. Seven strains of *C. botulinum* have been recognized on the basis of the antigenic specificities of their botulinum neurotoxins (BoNTs). Eight antigenic variants of botulinum neurotoxins (BoNTs) have been identified, A–G and X [2]. In Belgium, only 19 cases of foodborne botulism have been confirmed since 1988. Of these, 15 were identified as cases of type B botulism, one case as type A, and two cases for which neither the type nor the origin could be identified [3]. Foodborne botulism is a forgotten yet life-threatening disease.

## Case presentation

A Caucasian female in her 60s visited the emergency department (ED) with chief complaints of reflux with nausea and spasmodic epigastric pain, no vomiting, dry mouth, and weakness in both legs. These symptoms started around midnight. The lower limb weakness progressed overnight. In the morning, she alerted emergency medical services as she was unable to stand. She presented herself as a hemodynamically stable patient with bilateral ptosis, dysarthria, dysphagia, and decreased strength in both legs (grade 2/5), yet showed preserved strength in her upper limbs. She complained of light dyspnea with bilateral clear chest auscultation. Further clinical examination showed no particularities. During her stay in the ED, the patient complained of newly onset diplopia, gradually increasing difficulty of breathing, and weakness progressing to the upper limbs. An arterial blood gas was drawn and showed a type 1 respiratory insufficiency with hypoxia of pO_2_ 58 mmHg. Methemoglobin was within normal range. On further history, we learned that the patient consumed Atlantic wolffish with spinach, quinoa, and spring onions for dinner the night before. These were reheated leftovers from a meal prepared 2 days earlier. No particularities were found on biochemical and hematological investigations and computed tomography of the brain was normal. The patient deteriorated and needed to be intubated for respiratory support. Subsequently, she was admitted to the intensive care unit (ICU). Due to the suspicion of botulism, trivalent botulinum antitoxin (ABE antitoxin) was administered. Stool samples, stomach fluid, and blood samples were sent to the laboratory for testing on botulism toxins. Extract of the leftovers were injected in mice for observation and mouse bioassay. As primary differential diagnosis of Guillain–Barré syndrome was suspected, intravenous immunoglobulins (IVIG) were sequentially administered. Electromyography (EMG) on day 2 displayed an axonal motoric neuropathy. A spinal tap showed high immunoglobulin G (IgG) and high protein, but rather low cerebrospinal fluid (CSF)/serum albumin ratio. On day 4, the botulism toxins test, performed on stomach fluid and stool, confirmed the diagnosis of botulism. All the mice that were given the Atlantic wolffish died on day 3, whereas the mice that were given the fish and antitoxin were still alive. IVIG administration was discontinued. The patient was successfully extubated on the ninth day and left the ICU 4 days later. She made a full neurologic recovery and could leave the neurologic ward some days later. Afterwards, BoNT/E and *C. botulinum* type E were found during laboratory investigations. Five months later she is walking 14 km without problems and is starting back at her sports classes.

## Discussion

*Clostridium botulinum* is an anaerobic, gram-positive, spore-forming bacterium. Its growth is inhibited by an acidotic (pH less than 4.6) environment. If this condition is present, the bacillus will produce its toxin. If conditions are suboptimal, *C. botulinum* will produce spores for protection and conservation [4]. After ingestion, the toxin enters the bloodstream through the mucosa of the jejunum or ileum to finally disperse in the neuromuscular cholinergic synapse [5, 6]. In the peripheral cholinergic synapse, the toxin binds irreversibly with high selectivity and affinity to the presynaptic receptors. BoNTs bind selectively to motor neurons and autonomic cholinergic nerves, resulting in autonomous dysfunction characterized by bradycardia, mydriasis, dry mouth, and urinary retention [2, 7]. The spores are heat resistant; they can survive 100 °C for 5–6 hours. The toxin will be inactivated when heated above 85 °C for at least 5 minutes [5, 8].

The classic presentation of foodborne botulism is an acute onset of bilateral cranial neuropathies associated with symmetric descending weakness starting between 6 and 72 hours after ingestion [8]. Cranial nerve dysfunction includes blurred vision (secondary to fixed pupillary dilation and palsies of cranial nerves III, IV, and VI), diplopia, nystagmus, ptosis, dysphagia, dysarthria, and facial weakness. Descending muscle weakness usually progresses from the trunk and upper extremities to the lower extremities. Respiratory difficulties (for example, dyspnea) can be caused by diaphragmatic paralysis, upper airway compromise, or both, and often require intubation and mechanical ventilation [6, 9, 10]. Most patients present at the emergency department with predominantly gastrointestinal symptoms and less pronounced neurological changes [10, 11]. Other prodromal symptoms are dry mouth with sore throat [9]. A systematic review showed nausea was reported 36% of the time by patients with foodborne botulism and vomiting was reported 50% of the time. Cranial nerve involvement at admission was not reported for 7% of the patients. Among these, they were admitted with gastrointestinal signs and symptoms (for example, nausea, vomiting), with respiratory signs and symptoms (for example, shortness of breath), or with subjective weakness [10].

The diagnosis of botulism can be confirmed by the identification of toxins in serum, stool, vomitus, or in the food source. However, this detection requires multiple days for growth and identification of the organism [12]. *In vivo* testing from leftovers can be performed by IP injection of food extracted to mice. The decision to administer antitoxins should, however, be based on the presumptive clinical diagnosis and diagnosis should not delay therapy [6]. Antitoxin is the main therapeutic option for botulism. These antitoxins neutralize only circulating toxins; they have no effect on toxins already bound to the nerve terminals. Therefore, prompt administration early in the course of the disease is critical. O’Horo *et al*. concluded in their systematic review that antitoxin treatment reduces mortality. Earlier antitoxin administration reduces both mortality and ventilation time compared with later administration. However, they found no clear indication of a point in the course of illness at which antitoxin administration was no longer beneficial [13]. Equine serum heptavalent botulinum antitoxin (HBAT) contains antibodies (IgG) to seven of the eight known botulism toxin types (A through G). One vial (20 ml) should be slowly administered intravenously after dilution at 1:10 in normal saline. Starting infusion for at least 30 minutes at rate 0.5 ml/minute. If tolerated, infusion can be increased every 30 minutes at double rate until a maximum rate of 2 ml/minute [14]. In Belgium, only the trivalent botulinum antitoxin is available (Botulismus-Antitoxin Behring). It can only be provided by the government body Antigifcentrum and contains antibodies (IgG) to type A, B, and E [15]. Type A and B BoNT are the most frequent causative toxins in foodborne botulism, yet other toxins can be responsible. Two vials (250 ml) need to be administered slowly intravenously. A second dosage of a single vial should be administered after 4–6 hours [16]. No available evidence indicates that any particular patient characteristic (for example, age, sex, or preexisting health conditions) predicts better outcome from antitoxin administration. Patients with suspected botulism should be treated with BAT regardless of underlying medical conditions [6, 13]. Evidence does not indicate benefit from any treatment modalities other than antitoxin, although data are limited [13].

## Conclusion

Botulism can cause a variation of symptoms, often starting with predominantly gastrointestinal symptoms and less pronounced neurological changes. Rapid neurologic dysfunction and respiratory difficulties start between 6 and 72 hours after ingestion. The diagnosis of botulism can be confirmed by the identification of toxins in serum, stool, vomitus, or in the food source. However, this detection requires multiple days for growth and identification of the organism. The decision to administer antitoxins should, however, be based on the presumptive clinical diagnosis and diagnosis should not delay therapy. It is important to rapidly recognize the possible diagnosis of botulism even if neurological symptoms are not dominant.

## Data Availability

Not applicable.
